# Implementation of Integrated Care for the Aged Population in Anhui and Fujian Province of China: A Qualitative Study

**DOI:** 10.5334/ijic.6419

**Published:** 2022-06-10

**Authors:** Shasha Yuan, Meng Jia, Fang Wang

**Affiliations:** 1 Institute of Medical Information & Library, Chinese Academy of Medical Sciences & Peking Union Medical College, Beijing, 100020, China

**Keywords:** integrated care, implementation, aged population, qualitative study, China

## Abstract

**Introduction:**

Implementing integrated care for the aged population has been regarded as a mechanism to achieve healthy ageing. However, evidence from undeveloped nations has been scant. This study aims to explore the integrated care experience in Anhui and Fujian Province of China based on the Rainbow Model of Integrated Care (RMIC).

**Methods:**

The qualitative study was conducted in Anhui (in the middle area) and Fujian Province (in the eastern area) between May and September in 2018. The interviewees included twenty-eight policy makers working at departments of health and civil affairs at different levels and seventeen heads of medical and elderly care institutions.

**Results:**

The preliminary progress of integrated care in the sample cities of two provinces are mainly shown at solid policy basis by multiple key government agencies and political commitment achieved (system integration); preliminary coordination mechanism established between medical and elderly care institutions (organizational integration); consolidation of multi-disciplinary collaboration (professional integration); and reinforced role of family doctor teams for community-home dwelling elderly (service integration). Main challenges are also identified at insufficient inter-agency coordination, weak service capacity, lack of sustainable funding schemes, low level of information integration, and shortage of professional supply.

**Conclusion:**

Our findings provide a feasible path for other countries to strengthen integrated care for the aged population, particularly for those confronting rapid population ageing but with fragmented health care and elderly care systems.

## Introduction

The ageing of the population has become a global concern. Elderly over 65 years accounted for 9.3% of the total world population in 2020 and will reach 17% by 2050 [1]. In China, the latest seventh population census data reveals that elderly population with 65 years and older reached 190.6 million, accounting for 13.5% of total population in 2020 [2]. Meanwhile, the family size of Chinese elderly is shrinking as 13.1% of the elderly in China live alone, and only 38.2% live with their spouse reported by the latest survey in 2018 [3]. Due to high prevalence rate of non-communicable diseases, ageing presents increasing demand for both advanced elderly care and medical services [4]. Therefore, integrated care has been widely accepted as a feasible mechanism to achieve healthy ageing [5,6]. The World Health Organization (WHO) has developed series of reports to ensure the needs of older population being met by appropriately aligned health and long term care system, typically including “World Report on Aging and Health” [4], “Global Strategy and Action Plan on Aging and Health” [7] and “Integrated Care for Older People (ICOPE)” [8].

Integrated care is often contraposed to fragmented care and it is a challenging concept to define due to its multiple dimensions and varied scope [6,9]. Considering the ongoing reform in China, the term of integrated care in this study refers to the integration of medical services and elderly care. Since high-income countries have aged earlier, most of the evidence regarding integrated care are from Japan, Britain, America, the Netherlands, etc. For instance, laws or regulations are usually formulated to offer legal basis and to guide the development of integrated care for the elderly such as “the Old Age healthcare Law (1982)” in Japan [10] and “the National Health Service and Community Care Act 1990” in the United Kingdom (UK) [11]. In America, special programs such as “Programs of All-inclusive Care for the Elderly (PACE)” have been set to provide integrated care for the elderly including daily care, nursing care, preventative and medical services [12]. However, in undeveloped nations, the knowledge on integrated care was limited compared against dramatic growth of the absolute number of the elderly, which is unfavourable for the evidence-based policy making to achieve healthy ageing.

In China, the fragmentation between medical services and elderly care has resulted in huge inconvenience for the aged population [13]. Generally, medical services are mainly provided by primary healthcare institutions (PHIs) and hospitals at secondary or tertiary levels, which are supervised by National Health Commission (NHC). Elderly care institutions broadly included three types: elderly care home with overnight beds (long-term basis), daily care centre without overnight services, nursing home with registered nurses. They are under the supervision of Ministry of Civil Affairs (MCA). The independent structure of health care system and elderly care system aggravated the fragmentation between medical services and elderly care, including different requirements on infrastructure, service provision, personnel allocation, and fiscal funding [14]. Therefore, along with the deepening of ageing in China, strengthening the integrated care for the elderly residents has become a policy priority.

The term “integrated care (i.e., integrated medical services and elderly care)” was first mentioned in the document “Opinions to Accelerate the Development of Elderly care Industry” issued in September, 2013 [15]. Then, the General Office of China State Council (CSC) circulated a policy document “Guidelines to Promote the Integrated Care” in 2015 [16], which was jointly issued by the NHC, MCA and seven other related ministries. It is the first time in China to make integrated care towards aged population a national policy. Thereafter, many sectoral authorities have developed their own plans with specific priorities for the integrated care. Particularly, the NHC and MCA jointly published a document “Notice on Key Priorities of Relevant Ministries to Develop Integrated Care” in 2016 [17], which identified 36 tasks and mapped them to 18 government agencies. Under the guidance of afore-mentioned policies, local governments make explorative efforts for developing integrated care adapted to local context, which has generated preliminary valuable experience by now.

In this study, we aim to explore the implementation of integrated care for the aged population in the two provinces (Anhui and Fujian) of China based on the Rainbow Model of Integrated Care (RMIC) to enrich the academic evidence and offer some thoughts for other countries grappling with rapid population ageing and fragmented care.

## Theory and methods

### Theoretical framework

The provision of integrated care for the aged population is very complex, which involves integrative strategies at various levels and settings [6]. The RMIC was developed on the basis of literature review of various integrated care models, theories and by two international Delphi studies [18]. It defines integrated care from four dimensions (system integration, professional integration, organizational integration and service integration) and two enablers (functional integration and normative integration) [18] (see Supporting Text 1). The six key domains provide a framework to characterize the degree of integration from a multifocal perspective. The literature suggests that the RMIC has been well applied to explore and analyse the integration perspectives of different stakeholders’ at different levels in various study settings including Australia [19], Norway [20], Singapore [21], China [22,23,24,25], etc. Furthermore, Chen Z 2019 have already explored and validated the RMIC in China’s context, which confirmed its adaptation and applicability in China particularly regarding the key domains covered in this model [25]. Therefore, it is suitable to use RMIC as theoretical framework in this study.

### Study setting

Considering the economic status, demographic characteristics and the basis for health reform, Anhui province (in the middle area) and Fujian Province (in the eastern area) were selected to conduct this qualitative study. By the end of November in 2020 [2], the number of total population reached 61.02 million in Anhui Province with a rank of the 9th out of total 31 provinces (9/31) and 41.54 million in Fujian province (15/31). Elderly residents at 65 years and older accounted for 15.0% and 11.1% in Anhui and Fujian, respectively while it was 13.5% at the national level. The average disposable income in 2019 was 26415 RMB (around 4083 $) in Anhui Province and 35616 RMB (around 5505$) in Fujian Province.

### Data collection

The data was specifically collected in the two cities in Anhui Province (Hefei and Tongling city) and four cities in Fujian Province (Jinjiang, Zhangzhou, Longyan and Fuzhou city) between May and September in 2018. The interview protocol was designed based on the RMIC model focusing on specific integrative strategies and challenges encountered (see Supporting Text 2). The data was collected by individual interview and group interview, which were conducted face-to-face by three researchers (SY, FW, MJ) majoring in social medicine and health policy. Respondents were recruited until data saturation was reached [26,27]. To obtain a full understanding about the implementation of integrated care in China, the interviewees included policy makers at the departments of health and civil affairs at provincial, municipal, and county/district levels and heads of elderly care institutions, PHIs, and integrated care institutions. Here, integrated care institution refers to those transformed from original elderly care institutions or PHIs guided by the national policy, which can provide both medical services and elderly care.

Totally, twenty individual interviews (around 1 hours) and one group interview (around 2 hours) were conducted with policy makers; seventeen individual interviews (around 1 hours) were conducted with heads of related institutions (see Table 1). Additionally, we also collected relevant documents concerning integrated care, for instance, local policy and working reports, as supplementary materials for data triangulation.

**Table 1:** Respondents by level and location.


LEVEL	ANHUI	FUJIAN

Policy makers at departments of health and civil affairs		

Provincial	2	2

Municipal	2/8 (1)*	4

County/district	5	5

Heads of related institutions		

PHI	5	5

Elderly care institution	2	3

Integrated care institution	1	1

Total	25	20


* Notes group interview.

### Data analysis

The digital recordings were transcribed and cross-examined by two researchers (YS and JM). The data analysis follows a sequential approach of deductive and inductive steps [28]. The thematic analysis was adopted based on the analysis framework of the RMIC. Specifically, a codebook was developed based on the six RMIC domains (deductive): system integration, organizational integration, professional integration, service integration, functional integration and normative integration. Open labels (inductive) developed by YS and JM were used to identify relevant themes that emerged under each domain [28,29]. MAXQDA2018 (VERBI Software GmbH, Bismarckstraße 10–12, 10625 Berlin) and Excel 2016 were used to process the data. Triangulation in data analysis was achieved by comparing the interviews of different stakeholders and available policy documents and reports [27,28]. In addition to our narrative report of these findings, we also developed a summary table of integrative strategies organized by RMIC domains to make them visualized (Table 2).

**Table 2:** Preliminary progress and main challenges of integrated care based on RMIC.


RMIC	PRELIMINARY PROGRESS	MAIN CHALLENGES

System integration	– Solid policy basis: policy jointly issued by multiple government authorities. – Political commitment: the steering group on integrated care with the vice mayor as the leader.	Not mentioned.

Organizational integration	– Coordination among government agencies by joint policy release. – Inter-agency collaboration among medical institutions and elderly care institutions. – Collaboration between the government and social capital.	– Ineffective coordination among major stakeholders – Resistance of PHIs to transformed into integrated care institutions – Lack of initiative from social capital.

Professional integration	– In the form of multi-disciplinary collaboration – Typical examples: a) family doctor teams, neighbourhood committee, daily care staff and informal caregivers; b) physicians, nurses, and caregivers working at integrated institutions or at different medical and elderly care institutions.	– Multi-disciplinary collaboration is still very weak except for the integrated care institutions.

Service integration	– Greatly affected by organizational and professional integration. – The elderly living in the integrated care institutions enjoyed higher level of integrated care. – The family doctor contracting service has boosted the integrated care for community-home dwelling elderly.	– The weak capacity of PHIs cannot meet the increasing demand of home-based integrated care.

Functional integration	– Financing: financial budget (for beds, operation); covered by medical insurance. – Human resources: a) set up nursing programs and strengthening professional training; b) addressing the bottleneck of promotion; c) encouraging medical staff to practice at multiple institutions.	– Lack of sustainable funding scheme such as long-term care insurance – Low level and slow progress in information integration. – Professional shortage

Normative integration	– Social value: a) special attention and priority policies for the elderly in difficulty; b) advocacy of healthy ageing and elderly friendly environment.	– Ineffective integration of current independent service standards and lack of top-level design towards multi-disciplinary service standards.


## Results

### System Integration (at the macro-level)

#### Solid policy basis

Guided by national policies, local governments in Anhui and Fujian Province formulated local policy framework for the development of integrated care based on the features of local demographic characteristics and resources for medical services and elderly care. These policy documents formed the basis to stimulate integrated care for the aged population in practice. Representative policies issued by local governments are concluded in Supporting Text 3. For instance, in Anhui province, ten provincial departments jointly published “Implementation Guidelines for the Development of Integrated Care in Anhui” in 2016, which particularly emphasized that policy barriers among line ministries related to health, civil affairs, human resources and social security need to be removed to strengthen the synergies of supporting policies. In Fujian, provincial government makes integrated care be the priority of provincial health planning and formulated an action plan (2015–2020) to accelerate its development.

#### Political commitment

High-level political commitment was reported by the majority of respondents in the form of establishing a steering team chaired by the vice mayor. This steering team convenes multi-sectoral meetings and coordinates different responsibilities for new policy initiatives and implementation of pilots.

“Integrated care has become a top priority for our municipal government. The administrative and regulative power is much higher if the reform is led by government instead of single related department.” [health staff at municipal Department of Health]

### Organizational Integration (at the meso-level)

#### Multi-sectoral coordination

In practice, multi-sectoral coordination is usually in the form of joint policy release to define their responsibilities at policy level. Many important local documents to promote integrated care were jointly developed by Department of Health, General Office of Financial Stability Board, Department of Development and Reform, Department of Civil Affairs and Aging Committee, etc. For example, the close partnership between departments of health and civil affairs was particularly mentioned in Anhui:

“We share the same office building with Department of Health. We all love to attend each other’s agency meeting to discuss our common concern and find solutions. Information sharing and cooperation is not an issue for us.” [staff in municipal Department of Civil Affairs]

However, most interviewees indicated that they had difficulties in coordinating sectoral implementation of new policy initiatives. For example, it was typically reflected:

“Though new policies are jointly issued by several government agencies, it is still very challenging to operationalize the concept in practice, particularly regarding conflicting fire control standards for medical and elderly care institutions, fee schedule differences and different regulation requirement of insurance authorities.” [health staff at county Department of Health]

#### Collaboration between medical institutions and elderly care institutions

Three types of collaboration models can be concluded from the two provinces. First, elderly care institutions were encouraged to collaborate with the PHIs nearby to obtain timely primary health care guided by national and local policies. Service agreement was signed between the two types of institutions, which defined the contents and frequency of medical services provided and their responsibilities. Second, the PHIs and secondary hospitals with low bed utilization rate were stimulated to switch a certain percentage of beds for providing elderly care or to directly convert into nursing homes. However, the heads of PHIs indicated their resistance for such functional transformation due to unclear development in the future:

“PHIs and elderly care institutions follow different compensation and promotion systems in our province. There have been no clear standards or policy regarding our income and career development in the future”. [the head of PHI]

The third way is to set up medical department in elderly care institutions, which typically consists of one general practitioner (full-time or part-time), one pharmacist, and several nurses according to the size of the institution. They are equipped with necessary drugs (usually to treat chronic diseases like hypertension), medical and rehabilitation devices.

Noteworthy, the institutions in the second and third way had the advantage of medical resources including both professional and equipment so they can provide both medical services and elderly care. Therefore, they are called integrated care institutions and usually targets on the elderly with high medical demands such as disability, incapability and dementia.

#### Collaboration between governments and social capital

Public-Private Partnership (PPP) becomes more and more popular in the collaboration between governments and social capital regarding integrated care projects in China. The infrastructure was set up by local government through public funding and social capital were attracted to operate it. The contract was signed with detailed information regarding investment and responsibilities of each side. Meanwhile, local government usually offered preferential tax policies and waiving some fees for running integrated care institutions. However, at the initial stage, private investors kept cautious attitude in such integrated care projects due to unclear prospect and profit-driven nature.

“It is not easy to find a suitable private capital to run it. They preferred the high-end market segment (for rich elderly) instead of targeting the elderly with average income or lower income. It is contradicted with government goals.” [staff at provincial Department of Health]

### Professional Integration (at the meso-level)

Professional integration usually takes the form of multi-disciplinary collaboration among providers related to elderly care and medical services. For community-home dwelling elderly, multi-disciplinary collaboration involves members of the family doctor team, local neighbourhood committee, and informal-care givers. Given the close bond and strong trust between neighbourhood committee and local elderly, it is vital to collaborate with them to jointly provide primary health care. Currently, the collaboration between primary healthcare professionals and staff at daily care centre was more found in the form of lectures or health knowledge dissemination activities, which was still far from effective multi-disciplinary collaboration for service delivery.

Regarding institutional elderly, on the basis of organizational integration, general practitioners (normally called family doctors) and nurses at the cooperated PHIs came to visit elderly care institutions. Therefore, the elderly could enjoy both medical services and elderly care under such circumstance. In the case of integrated care institutions, the staff of medicine, nursing, rehabilitation and elderly care always work at the same institution to facilitate multi-disciplinary service delivery. For example, in one County of Fujian province, the beds for elderly care were newly set in the central PHI. The patients with geriatric conditions would be directly transferred to this centre after receiving rehabilitation care or medical services at the PHI, which typically represented multi-disciplinary collaboration of clinician, rehabilitation and elderly care professionals.

### Service Integration (at the micro-level)

Service integration is influenced by organizational and professional integration at the meso-level. Institutional elderly in an integrated care institution usually had better access to more integrated medical services and elderly care. Considering more than 90% of the elderly would live at home and community, providing integrated care for this group become priority for both Anhui and Fujian Province. The implementation of family doctor contracting service was regarded as a feasible way to promote the integration between elderly care and medical services. For instance, in one district of Anhui Province, a multi-disciplinary team (called “family doctor team”) consists of general practitioners, nurse, specialist from higher level hospital (member of the same medical alliance) and social worker. The family doctor team signs a service agreement with the elderly at home who are suffering from a particular chronic condition to provide targeted interventions. However, a typical problem is the huge gap between the ever-increasing great demand and limited service capacity of PHIs.

“Technical capacity of family doctor teams and limited health resources constrain the ability to offer integrated care for local elderly. Lack of policy support is another problem such as policy barriers for family beds for incapable elderly at home”. [the head of PHI]

### Functional integration

#### Financing arrangement

First, provincial fiscal funding was specifically set up to subsidize the provision of integrated care. The funding can be used to offer fix rate subsidy per bed or operational subsidy for integrated care providers. For example, Anhui Province gives a one-off construction subsidy of 2 000~5 000RMB (around 300~750$) per bed for private investors when they invest in an integrated care institution; Fujian Province offers an annual operational subsidy of no less than 20 000RMB (around 2 900$) for an urban community-based elderly care centre, and no less than 5 000 RMB (around 750$) for a rural township-based elderly care centre. Second, the integrated care providers who meet the eligibility criteria can become designated institutions of medical insurance. Therefore, medical services they provided could be reimbursed by medical insurance schemes.

However, the respondents indicated that these financing arrangements did not efficiently satisfy the needs of elderly residents. Lack of insurance coverage for related nursing services and elderly care was responded as bottleneck. Long-term care insurance has been commonly regarded as a feasible solution to the problem, which has not been established so far.

“The financing is highly fragmented for the named integrated care as medical services could be covered by medical insurance but daily care service was basically paid by out-of-pocket. For the elderly population (demand side), actually, there is little change in the financing model of integrated care.” [the head of elderly care institution].

#### Information Sharing

The interconnectivity of information systems between elderly care institutions and medical institutions is required to support information sharing for integrated care provision. At present, however, regarding health information system (HIS) itself, many medical institutions have poor interconnectivity with each other. Therefore, its connection with the information system of elderly care institutions is very limited. How to design and stimulate the information sharing among different service providers is a huge challenge which was frequently confronted by respondents. They indicated that national authority should have uniform planning for information sharing platform to guarantee its high operation efficiencies. Currently, Anhui Province is working on the development of a provincial smart healthcare platform that integrates all health-related data and elderly care information of local residents. This platform is based on the HIS and is as part of the national initiative “Internet+ healthcare services”.

#### Professional personnel cultivation

Ensuring sufficient professional personnel for the integrated care has been regarded essential to stimulate integrated care. Several strategies were implemented in the two provinces. First, colleges and universities were encouraged to set up nursing major, or add elderly care courses to the current curriculum. Meanwhile, more professional trainings were provided by local government. Second, the bottleneck in the promotion of health professionals for working at the integrated care institutions is being solved. They have been included in the same management system with those working at traditional medical institutions by health administration. Besides, they could enjoy some preferential policies in the professional title promotion, continuing education, and performance evaluation. Additionally, healthcare professionals are encouraged to practice in elderly care institutions and integrated care institutions as part-time worker to promote the mobility of professional resources between medical and elderly care institutions.

However, there is still a lack of professional personnel and training institutions. Most of the caregivers in the elderly care institutions and integrated care institutions are urban laid-off workers or migrant workers who are relatively not young and not well educated. Particularly, lack of professional nursing knowledge and skills has become a great obstacle for strengthening integrated care. The nursing beds are vacant in some institutions only because of lack of nursing staff.

“More than 1 300 seniors have been accommodated in the elderly care institutions in this city, and 232 staff are working in these institutions. After multiple trainings carried out by the departments of civil affairs and human resources, most of the staff have obtained primary certificates of licensure. However, their level of expertise and service quality are still not quite high”. [staff in municipal Department of Civil Affairs]

### Normative integration

#### Multi-disciplinary service standards

Multi-disciplinary service standards are vital for guide and regulate the quality of integrated care. Traditionally, medical service and elderly care are responsible by health authority and civil affairs authority respectively in China. Each authority has its own independent service standard and there is no national standard on the integrated care by the survey time.

“Currently, the elderly care institutions are the responsibility of the civil affairs authority, and the health authority is mainly in charge of improving medical services, which means these two functions are actually separated”. [staff in provincial Department of Health]

Although lack of national standard for integrated care, local service standards have already been formulated based on their piloting practice such as “Basic service specifications for the integrated care institutions” in Fujian province and “Manual on the standards of integrated care, home-based care and community care” in Anhui Province.

#### Social value

The social value towards aged population affects the implementation of integrated care. Both provinces have been fostering an enabling and friendly social atmosphere for the health of the elderly. Special attention and preferential policies were given to the integrated care for vulnerable groups. For example, Anhui Province prioritized the provision of integrated care to those elderly with low income, inability to work, or no supporters; and also subsidized the elderly care institutions that are willing to take in these seniors through government budget. In Fujian Province, multi-departments including provincial Communist Youth League Committee, Department of Civil Affairs, Aging Committee and Health Department have coordinated their work in raising the awareness on healthy aging and fostering friendly social atmosphere for aged population.

## Discussion

### Principal findings

Based on the RMIC model, this study explores the implementation of integrated care for the aged population in Anhui and Fujian Province, China. The preliminary progress has been shown in the joint release of integrated care policy by multiple key government agencies and political commitment achieved (system integration); preliminary coordination mechanism established between medical and elderly care institutions (organizational integration); enhancement of multi-disciplinary collaboration among medical staff, community workers, and caregivers (professional integration); and reinforced role of family doctor teams for community-home dwelling elderly (service integration). In the meanwhile, main challenges are also identified including insufficient inter-agency coordination, weak service capacity, lack of sustainable funding schemes, low level of information integration, and shortage of professional supply. Besides, related strategies at both functional integration (such as financing arrangement, information sharing and professional cultivation) and normative integration (such as multi-disciplinary service standards and social value) also played a vital role in the above-mentioned integration.

### Setting into international and evidence context

First, system integration is mainly shown at policy documents jointly issued by multiple relevant government agencies at the top-level design. It helps clarify the responsibility of key stakeholders and stimulate political commitment. However, in practice, lack of efficient inter-agency cooperation greatly hindered the implementation of integrated care policy. In China, the health system and elderly care system are independent from each other. The integration of medical services and elderly care also requires the coordination from other government agencies such as those in finance, medical insurance, human resource, and development and reform. Moreover, the related information regarding demographic, insurance coverage, and health information of the elderly is also scattered in different institutions and agencies, which further set obstacles for implementing integrated care policy. Additionally, although some national top-level design has been set in the arrangements of the integrated care, the policies in different fields such as capital input, infrastructure, technical support and professional training are unbalanced and lack specified rules [30].

Second, multi-disciplinary collaboration is closely related with the features of organizational integration and then affect service integration for the elderly at the micro level. The elderly living in the integrated care institutions usually receive more integrated care than those live at home and community or elderly care institutions. In the integrated care institution, the professional integration can be more effective as physicians, nurses and caregivers which constitute a multi-disciplinary team work at the same institution. On the contrary, multi-disciplinary teamwork between independent medical institution and elderly care institution still needs to be further improved. Furthermore, according to the national planning and culture factor, 90% of aged population in China is planned to live at home while only 3% is based on institutional care [31]. How to strengthen professional integration to provide integrated care towards community-home dwelling elderly is very vital, which is similar with the de-institutionalization in high-income countries. In China, family doctor contracting services implemented in 2016 has been considered as the most feasible way for the elderly at home and community to obtain integrated care [32]. However, the relatively weak capacity of family doctors gradually hinders the provision of integrated care for the community home based elderly and has become a major obstacle in the future reform [33].

Third, a sustainable funding scheme concerning integrated care has not been established yet in China. The current funding largely depends on government subsidy and social capital. Since the investment in integrated care institutions has long cycle and with unclear return, the profit-driven nature of private capital leads to the low engagement in the elderly care industry, thus restraining the development of the integrated care [34]. Moreover, the long-term care insurance which is prevalent in high-income countries are still in the piloting phase in China. Although some medical services and rehabilitation services can be reimbursed by medical insurance, the nursing service which accounts for a huge proportion in the integrated care for the elderly is still mainly paid by out of pocket [14]. The effectiveness of long-term care insurance including lowering medical cost for the elderly and reducing the burden for the home caregivers has been proven in many countries such as South Korea [35], Germany [36], and the Netherlands [37]. However, the evidence on the effects of long-term care insurance in developing countries has been very scant, which indicates the urgent need of further research. In China, Ministry of Human Resources and Social Security issued “Guiding Opinions on the Pilot Implementation of Long-term Care Insurance” in June 2016 [38], but systematic evidence regarding its effectiveness remains lacked.

Lastly, shortage of professional human resources in integrated care cannot meet the increasing demand of growing aged population. Our study reveals that both Anhui and Fujian Province have difficulties in recruiting the qualified staff for the integrated care institutions, especially professional nursing staff. According to the latest statistics from the WHO [39], the number of nursing personnel per 1 000 population is 1.655 in China while it is 9.884 in the US and 8.436 in the UK. On the one hand, the current nursing personnel in the labour market generally lack professional education to be qualified for the integrated care. On the other hand, low salary, unclear career development, and low social recognition also make the nursing job less attractive to recruit fresh graduates into this career. Similarly, the shortage of professional nursing staff is a common challenge for many high income countries [40]. Specific training standards, strict entry qualifications, and tiered training for elderly caregivers at different levels have been developed. For instance, the National Vocation Qualification (NVQ) system in the UK divides nursing staff into five ranks according to professional standard. Most of the care assistants working in the elderly care institutions have NVQ 2 level skills and takes 80% to 90% of the nursing work in those institutions [41]. These provide valuable lessons for China and other countries to develop their own training and education system for professional personnel in the integrated care.

### Strengths and limitations

This study provides comprehensive qualitative evidence regarding the implementation of integrated care for the aged population in Anhui and Fujian Province, China. It further enriches the academic evidence regarding integrated care from undeveloped nations. More importantly, the integrated care experiences in the two provinces provides a feasible path to achieve healthy ageing, which are particularly valuable for other countries with a fragmented health care system and elderly care system.

However, the study also has limitations. First, the property of this qualitative study determines that the findings of this study could not be generalized to other settings but other developing countries could benefit from the lessons indicated in this case study. Considering the limitation of qualitative study, a large-scale study with quantitative design or mixed study design regarding integrated care for the aged population should be carried out in the future to provide further empirical evidence support in China. Second, the demanding side (i.e., the elderly) is not included in the interviewees. The related information was mainly reflected by the heads of PHIs and elderly care institutions in this study. Therefore, the information regarding service integration at the micro-level might be bias. The views of demanding side should be included in the future research. Additionally, this research mainly focuses on qualitative analysis of the integrated care development in the two provinces based on RMIC. In the future, quantitative analysis using quantitative evaluation tools [42] can be conducted to further enrich the current research.

## Conclusion

The implementation of integrated care for the aged population in the sample cities in Anhui and Fujian Province of China has been promoted by solid multi-sectoral policy basis and political commitment at the macro-level, reinforced coordination mechanism and multi-disciplinary collaboration between elderly care and medical resources at the meso-level, and enhanced role of family doctor at the micro-level. It is also very essential to achieve functional integration regarding financing, information and professional cultivation and normative integration at multi-disciplinary service standards and social value. Our study suggests that future moves should be taken at inter-agency coordination, sustainable funding schemes, information integration, and capacity and supply of professional personnel. Moreover, a large-scale study on the integrated care for the aged population should be carried out to further provide empirical evidence in China.

## Lessons learnt

- Joint policy making by multiple key government agencies and achieving political commitment (system integration) provides the basis for implementing integrated care.
- The coordination mechanism between medical and elderly care institutions (organizational integration) and multi-disciplinary collaboration (professional integration) are essential to achieve integrated care, which are also tough in practice.
- Family doctor teams plays a vital role in the integrated care for community-home dwelling elderly (service integration).
- Supportive strategies at both functional integration (such as financing arrangement, information sharing and professional cultivation) and normative integration (such as multi-disciplinary service standards) are urgently needed.

## Disclaimers

We certify that the materials reported in this paper is not under consideration for publication elsewhere and its publication is approved by all authors.

## Additional Files

The additional files for this article can be found as follows:

10.5334/ijic.6419.s1 Supporting Text 1. Detrended Oscillation and Clock Parameters.

10.5334/ijic.6419.s2 Supporting Text 2. Interview protocols.

10.5334/ijic.6419.s3 Supporting Text 3. Representative policies on the integrated care in Anhui and Fujian Province.
