# Supplementary material for: Implementation of Integrated Care for the Aged Population in Anhui and Fujian Province of China: A Qualitative Study
Source: Int J Integr Care. 2022 Jun 10;22(2):20. doi: 10.5334/ijic.6419 (PMC9187242; doi:10.5334/ijic.6419)
Supplement: Supporting Text 1. — Detrended Oscillation and Clock Parameters. [file ijic-22-2-6419-s1.pdf]

Supporting Text 1 Integrated care dimensions of the Rainbow Model of Integrated Care (RMIC)

Level	Dimension	Definition
Macro leve	System integration	Refers to the alignment of rules and policies within a system.<br>A horizontal and vertical integrated system, based on a coherent set of (informal and formal) rules and policies between care providers and external stakeholders for the benefit of people and populations.
Meso level	Organizational integration	Refers to the extent to which organisations coordinate services across different organisations.<br>Inter-organisational relationships (e.g. contracting, strategic alliances, knowledge networks, mergers), including common governance mechanisms, to deliver comprehensive services to a defined population.
	Professional integration	Refers to extent to which professionals coordinate services across various disciplines.<br>Inter-professional partnerships based on shared competences, roles, responsibilities and accountability to deliver a comprehensive continuum of care to a defined population.
Micro level	Service integration	Refers to the extent to which care services are coordinated.<br>The coordination of person-focused care in a single process across time, place and discipline.
Linking the micro, meso and macro level	Functional integration	Refers to the extent to which back-office and support functions are coordinated.<br>Key support functions and activities (i.e. financial, management and information systems) structured around the primary process of service delivery, to coordinate and support accountability and decision making between organisations and professionals to add overall value to the system.
	Normative integration	Refers to the extent to which mission, work values etc. are shared within a system.<br>The development and maintenance of a common frame of reference (i.e. shared mission, vision, values and culture) between organisations, professional groups and individuals.

Adopted from Valentijn et al. (2013) (Valentijn PP, Schepman SM, Opheij W, et al. Understanding integrated care: a comprehensive conceptual framework based on the integrative functions of primary care. Int J Integr Care. 2013; 13(e010).)
