# Supplementary material for: Implementation of Integrated Care for the Aged Population in Anhui and Fujian Province of China: A Qualitative Study
Source: Int J Integr Care. 2022 Jun 10;22(2):20. doi: 10.5334/ijic.6419 (PMC9187242; doi:10.5334/ijic.6419)
Supplement: Supporting Text 2. — Interview protocols. [file ijic-22-2-6419-s2.pdf]

Supporting Text 2 Interview protocols

For policy makers at the departments of health and civil affairs

1. Please give a general introduction to the integrated care reform from the perspectives of the overall design, ideas, announced relative documents, implementation plan and so on.
2. Could you specify how to practice multi-sectoral coordination during implementing integrated care?
   a) For the health authority: What is the coordination mechanisms among the departments of interests, such as Civil Affairs Bureau, Medical Insurance Department, Finance Department, and Department of Human Resources and Social Security?
   b) For the civil affairs authority: What is the coordination mechanisms among the departments of interests, such as Department of Health, Department of Human Resources and Social Security, Finance Department, etc?
3. Please introduce in detail how the local medical institutions get involved in the provision of integrated care, especially the participation model and coordination mechanism of primary healthcare institutions.
4. Please introduce in detail how integrated care are provided to the elderly living at home and community, and in elderly care institutions, respectively, such as specific service providers, service models, service charges, and primary effects.
5. Please introduce in detail about the security mechanism for integrated care, particularly regarding financing, professional cultivation, and resources (information) sharing system and so on.
6. What supports are still needed in order to develop effective integrated care in the future?

For heads of primary healthcare institutions

1. Please introduce the basic situation of the elderly and the resource distribution of medical care and elderly care in your jurisdiction?
2. Please elaborate the practices of the integrated care provided by your institution, such as specific service contents, family beds, setting up elderly care center, etc.
3. How does your institution provide the medical service to the home-community dwelling, particularly the role of family doctor team playing in the integrated care?
4. How do you feel when you are engaged in medical treatment, nursing care and rehabilitation for the elderly? What are the incentive mechanisms for the medical staff in your institution to provide elderly care?
5. Please specify how your institution cooperate with other elderly care institutions in your jurisdiction and how the professionals from different fields coordinate with each other.
6. Can the current situation of resource allocation (staff, beds, and nurses) meet the demands of integrated care for the elderly?
7. What are the main problems to further implement the integrated care for the elderly?

For heads of elderly care institutions

1. Please introduce the basic situation of your institution, including staff and bed allocation, available services and so on.
2. Please talk about the basic situation of the elderly in your institution, especially about their health status and demands for medical services.
3. Please introduce in detail the policy supports your institution receive during the implementation of integrated care, for instance, financial supports or land approval?
4. Please specify how your institution cooperate with medical institutions and how the professionals from different fields coordinate with each other.
5. Do you think the current services provided can satisfy the demands of the elderly living in your institutions? If not, what are the deficiencies?
6. What challenges are you confronting at present? Could you tell how to solve them?

For heads of integrated care institutions

1. Please introduce the basic situation of your institution, including staff and bed allocation, available services and so on.
2. Please introduce the basic situation of the elderly living in your institution, especially about their health status and demands for medical services and elderly care.
3. Please introduce in detail the policy supports your institution receive during the implementation of integrated care, for instance, financial supports or land approval?
4. Pleases specify the provision of integrated care in your institution, for example, the involved personnel, service contents, charges, etc.
5. Please describe in detail how the multi-disciplinary personnel work together in your institution such as the coordination between medical staff and elderly care givers.
6. Could the current situation of resource allocation (staff, beds, information, etc) meet the demands of integrated care for the elderly? If not, what are the deficiencies?
7. What challenges are you confronting at present? Could you tell how to solve them?
