# Supplementary material for: Implementation of Integrated Care for the Aged Population in Anhui and Fujian Province of China: A Qualitative Study
Source: Int J Integr Care. 2022 Jun 10;22(2):20. doi: 10.5334/ijic.6419 (PMC9187242; doi:10.5334/ijic.6419)
Supplement: Supporting Text 3. — Representative policies on the integrated care in Anhui and Fujian Province. [file ijic-22-2-6419-s3.pdf]

Supporting Text 3 Representative policies on the integrated care in Anhui and Fujian Province

Location	Policy released	Issued authorities	Main Contents
Anhui	Guidelines to promote the integrated care.	Health Commission, Civil Affairs Bureau, Commission of Development and Reform, Department of Finance, Commission of Human Resource and Social Security, Department of Natural Resources, Department of Housing and Urban-Rural Development, Department of Commerce, Committee on Aging, Department of Traditional Chinese Medicine of Anhui Province	A programmatic document on the integrated care in "13th Five-Year Plan" period clearly points out the work objectives, primary mission, guarantee measures, which calls for "breaking down the policy barriers among health department, civil affair administration, human resources and social security department, national land department" and "Taking the establishment of integrated care project as a key to promote the deep integration of medical institutions and elderly care institutions throughout the province."
	Action plan on constructing a multi-level elderly care system (2018-2020) in Anhui Province.	The General Office of the Government in Anhui Province	Strengthen the main suppliers of integrated care, enhance the health management of the elderly, and promote the basic medical system and long-term care insurance system.
	Guidelines for developing the smart health in the integrated care.	Civil Affairs Bureau, Health Commission, Committee on Aging	A multi-level and diverse smart integrated care system should be basically formed by 2020 with the significant improvement in elderly care, medical services, sports and entertainment, spiritual comfort and so on.
	Implementation opinions on fully opening-up the elderly care market and improving the quality of elderly care.	The General Office of the Government in Anhui Province	The service network of community-home dwelling elderly in urban and rural areas should be basically formed with no less than 70% of aged care beds and no less than 30% of nursing beds operated by social capital.
Hefei City in Anhui Province	Opinion on piloting implementation of including elderly care institutions providing integrated care into	Hefei Human Resources and Social Security Bureau	It clearly regulates in policies about the inclusion criteria of elderly care institutions providing integrated care as designated institutions of medical insurance, the conditions of the elderly reimbursed by

	designated institutions of medical insurance.		basic medical insurance, the subjects of nursing, payment methods and the management of designated institutions covered by medical insurance.
	The use and management regulations on the support funds of financial department for the construction of community elderly care facilities in Hefei City.	Department of Finance and Civil Affairs Bureau of Hefei	Make up for weakness of home-based elderly care in both urban and rural areas; greatly improve the quality of integrated care for the community-home dwelling elderly.
Tongling City in Anhui	Implementation plan on improving home-and-community based health security and further promoting the pilot program of integrated care.	Tongling Health Commission	Expand the pilot scope of the integrated care.
	Handbook for integrated care and home-and-community based care in Tongling City.	Tongling Health Commission and Civil Affairs Bureau	Specify related service standards for the integrated care.
Fujian	Opinions on promoting the implementation of integrated care.	The General Office of the Government of Fujian Province	Clarify the principles, objectives, tasks, and measures of the integrated care in Fujian.
	Action plan on accelerating the development of health and aging service project (2015-2020) in Fujian.	Commission of Development and Reform, Health Commission, Education Department, Civil Affairs Bureau, Department of Finance, Sports Administration of Fujian Province	Clarify priority in the three areas concerning the health service system, the elderly care system and the construction of facilities for physical fitness; clarify 158 major projects of health and elderly care in 2017 and 161 in 2018, accounting for CNY 54.35 billion and CNY 49.3 billion in total separately.
	Implementation plan on strengthening reforms to streamline the government, delegate the power, and improve government	Health Commission of Fujian Province	Abandon the approval on clinic operation within the elderly care institutions and adopt a record-reporting system; Integrate the approval for the registration of medical institution and practice of medical service into one process for medical institutions at

	services for inspiring the vigor in health field.		secondary level or below; and encourage elderly integrated care institutions to establish the medical department or set up clinics or nursing station within the institutions in accordance with different institution scales and actual demands.
Zhangzhou City in Fujian	Notice on selecting pilot institutions for the integrated care.	Department of Health and Civil Affairs Bureau of Zhangzhou City	According to actual practices, every county, city or district is required to apply for municipal pilot institutions for the integrated care. Priority includes: integrated care institutions, contract-based collaboration between elderly care institutions and medical institutions, integrated care specifically for community-home dwelling elderly, making full use of vacant beds in primary healthcare institutions and daily care centers, etc.
	Notice on promoting the collaboration agreement between medical institutions and elderly care institutions.	Department of Health and Civil Affairs Bureau of Zhangzhou City	It is required that all the elderly care institutions should sign cooperation agreements with the nearby medical institutions at the same level. Professional medical services should be provided to the elderly with the clear definition on the responsibilities, rights and obligations, services items and approaches of both sides, the cooperation mechanism for emergency treatment and hospital transfer.
